# Effective elimination of adult B-lineage acute lymphoblastic leukemia by disulfiram/copper complex *in vitro* and *in vivo* in patient-derived xenograft models

**DOI:** 10.18632/oncotarget.9413

**Published:** 2016-05-17

**Authors:** Manman Deng, Zhiwu Jiang, Yin Li, Yong Zhou, Jie Li, Xiangmeng Wang, Yao Yao, Weiguang Wang, Peng Li, Bing Xu

**Affiliations:** ^1^ Department of Hematology, The First Affiliated Hospital of Xiamen University, Xiamen, China; ^2^ Key Laboratory of Regenerative Biology, Southern China Institute for Stem Cell Biology and Regenerative Medicine, Guangzhou Institutes of Biomedicine and Health, Chinese Academy of Sciences, Guangzhou, China; ^3^ Department of Hematology, Nanfang Hospital, Southern Medical University, Guangzhou, China; ^4^ Drug Discovery Pipeline, Guangzhou Institutes of Biomedicine and Health, Chinese Academy of Sciences, Guangzhou, China; ^5^ Research Institute for Healthcare Science, Faculty of Science and Engineering, University of Wolverhampton, Wolverhampton, UK

**Keywords:** disulfiram, copper, adult B-cell acute lymphoblastic leukemia, p16 deletion, patient-derived xenograft

## Abstract

Disulfiram (DS), a clinically used drug to control alcoholism, has displayed promising anti-cancer activity against a wide range of tumors. Here, we demonstrated that DS/copper (Cu) complex effectively eliminated adult B-ALL cells *in vitro* and *in vivo* in patient-derived xenograft (PDX) humanized mouse models, reflected by inhibition of cell proliferation, induction of apoptosis, suppression of colony formation, and reduction of PDX tumor growth, while sparing normal peripheral blood mononuclear cells. Mechanistically, these events were associated with disruption of mitochondrial membrane potential and down-regulation of the anti-apoptotic proteins Bcl-2 and Bcl-xL. Further analysis on B-ALL patients' clinical characteristics revealed that the *ex vivo* efficacy of DS/Cu in primary samples was significantly correlated to p16 gene deletion and peripheral blood WBC counts at diagnosis, while age, LDH level, extramedullary infiltration, status post intensive induction therapy, immune phenotype, risk category, and Ph chromosome had no effect. Together, these findings indicate that disulfiram, particularly when administrated in combination with copper, might represent a potential repurposing agent for treatment of adult B-ALL patients, including those clinically characterized by one or more adverse prognostic factors.

## INTRODUCTION

Acute lymphoblastic leukaemia (ALL) is a clinically and biologically heterogeneous disorder [1, 2]. Despite the introduction of the first-line therapy, including high-dose multi-agent combination chemotherapy (increasingly inspired to pediatric principles), hematopoietic stem cell transplantation, and new targeted therapy, which has significantly improved overall survival rate (approximately 85%) of pediatric ALL patients, only about 30-40% of adults with ALL achieve long-term disease-free survival (DFS) [3–5]. Among others, severe adverse events that threaten the lives of adult, especially elderly, patients with ALL represent the major hindrances to the high-dose multi-agent combination chemotherapy regimens. Therefore, new therapeutic approaches with high efficacy but low toxicity are urgently needed to treat adults with ALL, in order to improve their long-term DFS as well as overall survival.

Disulfiram (DS), a member of the dithiocarbamate family, is an FDA-approved drug that has been clinically used as an alcohol-abuse deterrent for more than six decades [6, 7]. In contrast to conventional chemotherapy agents, it exhibits low toxicity, while easily available and inexpensive. DS, as a strong metal-ion chelating agent, interacts with copper (Cu) to form the Ds/Cu complex with enhanced anti-tumor activity [8–10]. Recently, several studies have demonstrated that DS is highly effective against various types of solid tumors such as breast cancer [11–13], melanoma [9, 14, 15], and prostate cancer [16], as well as hematological malignancies, including ALL [17–19].

Apoptosis, known as type I programmed cell death, plays a critical role in development and homeostasis of organisms [20, 21]. There are two major apoptotic signaling cascades, the mitochondria-mediated intrinsic pathway and the death receptor-mediated extrinsic pathway. The former is often considered as the classic apoptotic pathway, which is initiated with mitochondrial injury (e.g., loss of mitochondrial membrane potential), resulting in mitochondrial outer membrane permeabilization (MOMP) and thus release of cytochrome c from mitochondria to cytoplasm where it forms apoptosome with Apaf-1 to cleave/activate caspase 9, followed by cleavage/activation of the executioner caspase-3, ultimately inducing apoptosis [22, 23, 24].

Currently, it remains to be defined whether and by what mechanism(s) DS/Cu would be active against adult B-lineage acute lymphoblastic leukemia (B-ALL). Here we report that DS/Cu is significantly and selectively cytotoxic *in vitro* against human B-ALL cell lines and primary samples obtained from adults with B-ALL, particularly those carrying adverse prognostic genetic abnormalities (e.g., p16 deletion), as well as effective *in vivo* in B-ALL patient-derived xenografts, in association with activation of the intrinsic apoptotic pathway, at least in part, due to down-regulation of Bcl-2 and Bcl-xL.

## RESULTS

### DS/Cu exhibits dose-dependent cytotoxicity in human B-lineage acute lymphoblastic leukemia cell lines

**Figure 1:**
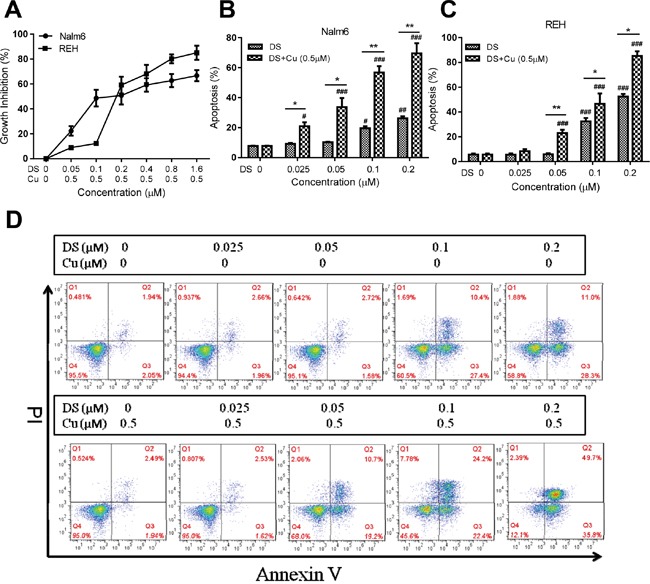
DS/Cu displays dose-dependent cytotoxic effects on human B-ALL Nalm6 and REH cells **A.** Nalm6 and REH cells were exposed to the indicated concentrations of DS in the presence of 0.5 μM Cu for 24 hr, after which anti-proliferative effect was determined by a CCK-8 kit. **B, C.** Nalm6 (B) and REH cells (C) were treated with a series of concentrations of DS with or without 0.5 μM Cu for 24 hr, after which percentage of apoptotic cells was determined by flow cytometry using Annexin V/PI double staining. ^#^ or * *P*<0.05, ^##^ or ** *P*<0.01, ^###^ or *** *P*<0.001 vs untreated control or DS alone, respectively. **D.** Representative data for flow cytometric analysis of Annexin V/PI staining in Nalm6 cells after exposed (24 hr) to the indicated concentrations of DS with or without Cu (0.5 μM).

First, we examined the cytotoxic effect of DS/Cu on two human B-ALL cell lines (i.e., Nalm6 and REH) using the Cell Counting Kit-8 (CCK-8). As shown in Figure 1A, while treatment with Cu alone had no significant effect on cell proliferation (inhibition rate=6.39±4.93%, *t*=-2.244, *P*=0.154 vs untreated control; not shown), exposure to a series of the indicated concentrations of DS coupled with 0.5 μM Cu (DS/Cu) for 24 hrs significantly inhibited proliferation of Nalm6 cells in a dose-dependent manner (*P*<0.05 for each condition vs untreated control), with IC50 values of 0.18±0.08 μM. Analogous results were obtained in another B-ALL cell line, REH (Figure 1A; *P*<0.05 for each condition vs untreated control), with IC50 values of 0.243±0.256 μM.

We next examined whether DS with or without Cu induce apoptosis in B-ALL cell lines. To this end, after exposed to various indicated concentrations of DS in the presence or absence of 0.5 μM Cu for 24 hrs, flow cytometric analysis with Annexin V/PI double staining was performed to determine the percentage of apoptotic cells. In both Nalm6 and REH cells, Cu (0.5 μM) administrated alone was unable to induce apoptosis (*P*>0.05 vs untreated control; see below Figure 1D). However, whereas ≥0.1 μM DS alone had significant effects, treatment with DS at different doses (0.025, 0.05, 0.1, 0.2 μM) in combination with 0.5 μM Cu for 24 hrs resulted in significantly increased apoptosis in a dose-dependent manner in Nalm6 cells (Figure 1B; *P*<0.05 for each DS dose, DS/Cu vs either untreated control or DS alone). Comparable phenomena were observed in REH cells (Figure 1C; *P*<0.05 for each DS dose, except 0.025 μM, DS/Cu vs untreated control or DS alone). In addition to marked increases in overall percentage of Annexin V^+^ apoptotic cells when compared DS/Cu with DS alone, representative flow cytometric data also demonstrated that whereas DS (≥ 0.1 μM) by itself predominantly induced early apoptosis (Annexin V^+^/PI^−^, lower/right quadrant Q3; Figure 1D, top panels), co-treatment (24 hrs) with DS (≥ 0.05 μM) and Cu (0.5 μM) resulted in robust increases in both early and late apoptosis (Annexin V^+^/PI^+^, upper/right quadrant Q2; Figure 1D, bottom panels). However, exposure to neither DS nor DS/Cu resulted in necrosis (Annexin V^−^/PI^+^, upper/left quadrant Q1). Taken together, these results indicate that whereas DS itself is active against B-ALL cell lines, combined administration with non-toxic concentrations of Cu (e.g., 0.5 μM) remarkably potentiates cytotoxicity of DS, primarily via induction of apoptosis in a dose-dependent manner.

### DS/Cu preferentially induces apoptosis of primary adult B-ALL cells, but not normal PBMCs

**Figure 2:**
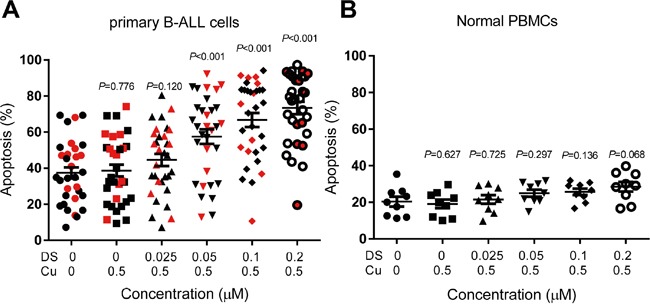
DS/Cu induces apoptosis in primary adult B-ALL cells but not normal PBMCs **A, B.** mononuclear cells isolated from primary bone marrow of adults with B-ALL (A) and peripheral blood of healthy donors (B) were exposed to the indicated concentrations of DS with 0.5 mM Cu for 24 hr, after which flow cytometric analysis was performed to determine percentage of Annexin V^+^ cells. The patients carrying p16 gene deletion are highlighted by red symbols. Horizontal lines represent the mean and SD of the values obtained from 32 patients (A) and 9 healthy donors (B).

We then tested activity of DS/Cu in primary samples (bone marrow mononuclear cells) obtained from adults with B-ALL. Clinical characteristics of these B-ALL patients are summarized in Table 1 (also see Supplemental Table S1 for details). Consistent with the anti-leukemia activity of DS/Cu observed in B-ALL cell lines, co-treatment (24 hrs) with ≥ 0.05 μM DS and 0.5 μM Cu resulted in significant increases in apoptosis of primary B-ALL cells (*P*<0.001 vs untreated control, n=32; Figure 2A), although the responses varied among patients. Regardless of the marked differences in basal levels of spontaneous cell death among these primary samples, average net increases in percentage of apoptotic cells were 7.16% 20.14% 29.52% 36.01% for 0.025, 0.05, 0.10 and 0.20 μM DS in combination with 0.5 μM Cu, respectively, while only 1.29% for Cu alone (Table 2). Of note, identical treatments with DS/Cu displayed minimal toxic towards normal peripheral blood mononuclear cells (PBMCs) obtained from healthy donors of hematopoietic stem cell transplantation (HSCT, Figure 2B and Table 2). These findings argue that DS/Cu might selectively eliminate B-ALL cells, while sparing normal hematopoietic cells, in consistence with low-toxicity of DS as a safe anti-alcoholism drug.

**Table 1:** Patient clinical characteristics (n=32)

Characteristic	Value	Number of patients	%
Age, years			
Median (range)	27.5 (15-61)		
≥35		11	34.4
<35		21	65.6
WBC(×10^9^/L) Median (range)	46.3 (1.3-429.5)		
≥30		22	68.8
<30		10	31.2
LDH			
High		22	68.8
Normal		10	34.4
Status of d14			
CR		20	62.5
NR		12	31.2
Status after induction therapy			
CR		25	78.1
NR		7	21.9
Extramedullary infiltration			
Yes		24	75
No		8	25
Immune phenotype			
Pro-B		13	40.6
Non-proB		19	59.4
Risk category			
High		13	40.6
Standard		19	59.4
Ph chromosome			
Positive		11	34.4
Negative		21	65.6
p16 gene deletion			
Positive		12	42.9
Negative		16	57.1

Abbreviations: WBC, white blood cell; LDH, lactic dehydrogenase; CR, complete remission; NR, No remission.

**Table 2:** Effects of DS treatment with or without Cu on human primary adult B-ALL cells (n=32) and normal PBMCs (n=9)

DS (μM)	0	0	0.025	0.05	0.10	0.20	*P*- value
Cu (μM)	0	0.5	0.5	0.5	0.5	0.5	
Primary B-ALL cells	37.36±17.49	38.65±18.68	44.52±18.84	57.50±22.87	66.88±21.68	73.37±19.20	0.000
Normal PBMCs	19.57±7.40	17.87±7.14	20.89±8.22	23.04±6.18	24.76±6.57	26.82±8.26	0.117

Primary B-ALL cells and normal PBMCs were incubated with the indicated doses of DS in the presence or absence of Cu for 24hr, after which percentage of apoptotic cells were assessed by flow cytometry. Values represent mean ± SD for at least three independent experiments.

### Cytotoxicity of DS/Cu against primary B-ALL cells correlates to p16 deletion and WBC count at diagnosis in adults with B-ALL patients

To examine whether the clinical features of adult B-ALL patients (Table 1 and Supplemental Table S1) would have any effects on anti-leukemia activity of DS/Cu against primary B-ALL cells, we analyzed the potential relationship between patients' characteristics and the percentage of apoptotic cells induced by a series of concentrations of DS in combination with 0.5 μM Cu. According to the NCCN Guidelines Version 1.2015 Acute Lymphoblastic Leukemia, high risk is generally defined as having any of the following poor-risk cytogenetic factors: hypodiploidy (<44 chromosomes); t(v;11q23) or MLL rearrangements; t(9;22) or BCR-ABL gene mutations; or complex karyotype (≥5 chromosomal abnormalities), while the absence of all poor-risk factors is considered standard risk.

Of total 32 patients enrolled in this study, median age was 27.5 years (range, 15-61 years); 11 (34.4%) were age ≥ 35 years; median peripheral white blood cell (WBC) count at diagnosis was 46.30 × 10^9^/L, (range, 1.27-429.46 × 10^9^/L); and 13 (40.6%) and 19 (59.4%) had high or standard risk diseases, respectively. As shown in Table 3, two-way ANOVA analysis revealed that the *ex vivo* efficacy of DS/Cu towards primary B-ALL cells was significantly associated with WBC count at diagnosis (*P*=0.044) and p16 gene deletion of patients (*P*=0.008). However, other clinical characteristics (e.g., age, LDH levels, extramedullary infiltration, status at 14^th^ and 28^th^ day post intensive induction therapy, immune phenotype, risk category, Ph chromosome, etc.) did not significantly affect response of primary B-ALL cells to DS/Cu (*P*>0.05 for each of these parameters). These results raise a possibility that adults with B-ALL carrying certain adverse prognostic genetic abnormalities (e.g., p16 gene deletion) might be particularly susceptible to the DS/Cu regimen.

**Table 3:** The relation between clinical characteristics of B-ALL patients and *ex vivo* cytotoxicity of DS/Cu in primary samples

Characteristic	DS(μM)	0	0	0.025	0.05	0.10	0.20	*P*- value
	Cu(μM)	0	0.5	0.5	0.5	0.5	0.5	
Age, years	≥35(n=11)	37.36± 17.91	38.11± 18.53	45.47± 19.96	57.43± 23.71	65± 23.59	72.81± 20.61	
	<35(n=21)	37.35± 17.49	39.68± 19.81	42.71± 17.26	57.62± 22.31	69.88± 18.09	74.44± 17.06	0.744
WBC(x10^9^/L)	≥30(n=22)	40.04± 19.37	41.96± 20.28	48.88± 18.27	62.57± 21.65	69.26± 21.59	73.88± 19.09	
	<30(n=10)	32.82± 11.31	35.48± 14.53	39.55± 16.84	51.46± 24.1	61.88± 22.32	69.09± 18.83	0.044*
LDH	High(n=22)	38.80± 18.95	40.52± 19.92	44.67± 22.28	55.83± 25.61	64.37± 23.25	71.77± 20.82	
	Normal(n=10)	34.61± 15.85	36.13± 17.63	48.62± 14.92	62.6± 16.43	71.96± 18.04	76.02± 15.26	0.644
Status of d14	CR(n=20)	37.17± 17.7	38.91± 18.66	45.97± 18.15	56.3± 23.19	63.92± 20.85	71.80± 17.4	
	NR(n=12)	36.7± 19.76	38.41± 21.32	42.71± 21.97	57.39± 25.91	69.62± 23.23	74.32± 21.83	0.577
Status after induction therapy	CR(n=25)	40.42± 17.46	39.88± 17.98	46.83± 17.52	59.63± 22.7	67.43± 20.67	73.94± 18.35	
	NR(n=7)	33.86± 21.82	35.95± 24.92	38.55± 24.63	50.6± 25.48	63.76± 28.04	69.52± 23.8	0.106
Extramedullary infiltration	yes(n=24)	37.42± 16.94	38.89± 17.84	42.24± 18.73	55.95± 21.15	65.73± 22.72	73.42± 20.39	
	No(n=8)	37.7± 21.79	39.91± 23.69	54.41± 17.26	63.89± 15.05	69.78± 19.68	72.13± 15.78	0.542
Immune phenotype	Pro-B(n=13)	36.35± 19.94	37.46± 20.5	45.34± 17.69	62.97± 17.7	71.11± 16.46	75.58± 13.25	
	Non-proB(n=19)	38.28± 16.86	40.30± 18.49	45.24± 20.12	54.49± 26.04	63.75± 24.73	71.4± 22.46	0.484
Risk category	High(n=13)	39.25± 19.75	42.03± 20.95	45.21± 19.13	58.6± 19.06	69.34± 18.59	75.11± 17.13	
	Standard(n=19)	36.29± 16.96	37.17± 17.97	45.32± 19.22	57.44± 26.05	65.08± 23.75	71.28± 22.03	0.341
Ph chromosome	Positive(n=11)	41.18± 20.94	44.19± 21.86	44.53± 19.95	55.84± 19.27	66.39± 18.74	72.48± 17.39	
	Negative(n=21)	35.56± 16.3	36.51± 17.4	45.67± 18.77	59.03± 25.23	66.93± 23.63	73.44± 20.39	0.994
p16 gene deletion	Positive(n=12)	41.41± 15.02	45.39± 17.63	48.56± 17.34	61.28± 24.65	67.27± 26.21	73.71± 22.52	
	Negative(n=16)	30.09± 16.49	30.37± 16.57	38.08± 18.07	52.57± 23.43	65.06± 20.38	72.04± 18.69	0.008**

Primary B-ALL cells were exposed to the indicated concentrations of DS with or without Cu for 24hr, after which percentage of apoptotic cells were determined by flow cytometry. Values represent mean± SD of at least three independent experiments. *P<0.05, **P<0.01.

### DS/Cu suppresses colony formation of B-ALL cells

**Figure 3:**
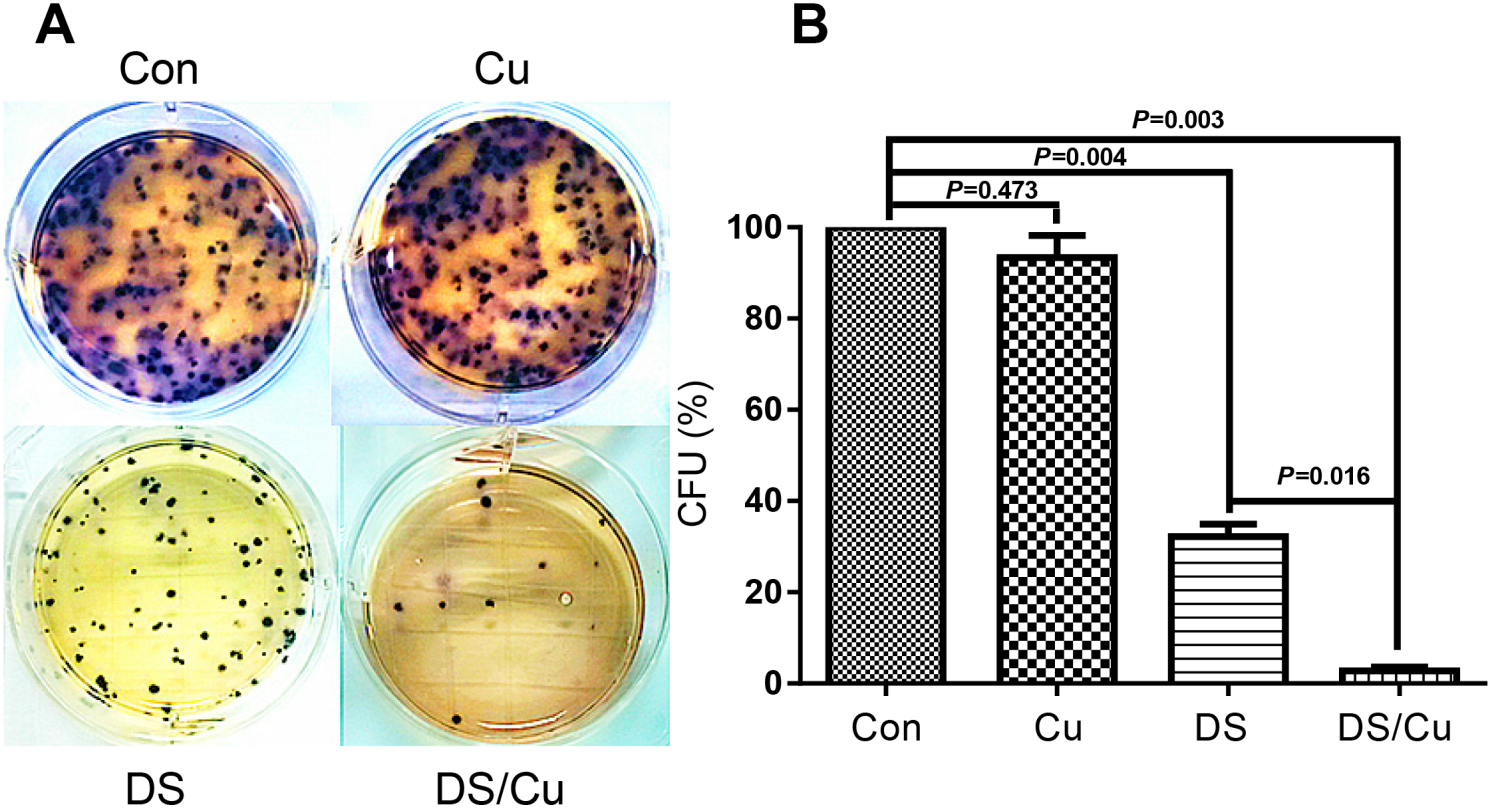
DS/Cu significantly abolishes the colony-forming ability of Nalm6 cells **A.** Nalm6 cells were exposed to 0.05 μM DS with or without 0.5 μM Cu for 6 hr, after which clonogeic assay was performed as described in Methods. **B.** Percentage of colony-forming units (CFU) was determined by counting colonies (≥50 cells). Values represent the mean ± SD for at least three independent experiments.

Clonogenic assays were carried out to investigate the ability of DS/Cu to induce “reproductive death” in B-ALL cells. As shown in Figure 3, while treatment with Cu (0.5 μM) had no effect (*P*>0.05 vs untreated control), treatment (6 hrs) with DS (0.05 μM) alone markedly reduced colony number in Nalm6 cells (*P*<0.01 vs untreated control). Of note, combined treatment with DS and Cu (6 hr prior to plating) almost completely abolished the colony-forming ability of Nalm6 cells after cultured for 10-14 days (*P*<0.01 and *P*<0.05 vs untreated control and DS alone, respectively). These results indicate that DS is able suppress clonogenicity of B-ALL cells, an event dramatically enhanced by co-administration of Cu.

### DS/Cu induces loss of mitochondrial membrane potential and activation of the intrinsic apoptotic pathway, in association with down-regulation of Bcl-2 and Bcl-xL

**Figure 4:**
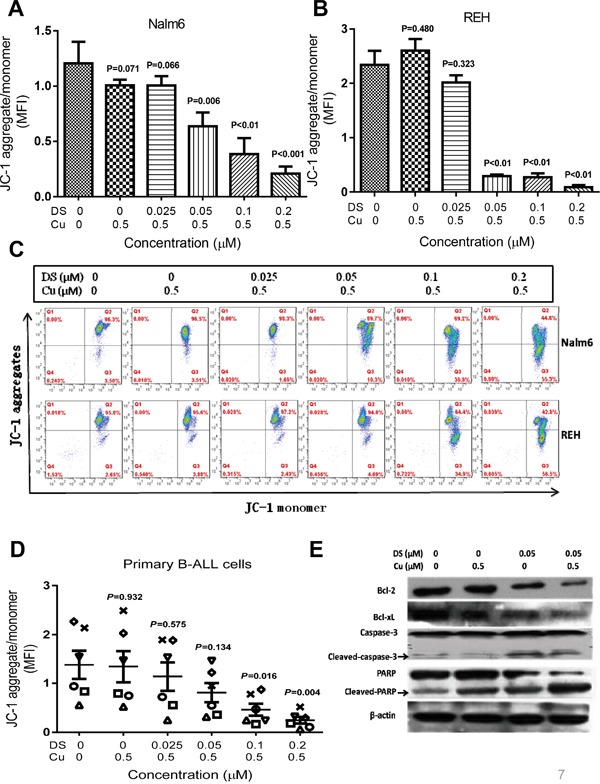
DS/Cu activates the mitochondria-related intrinsic apoptotic pathway in B-ALL cell lines and primary cells **A-D.** Nalm6 (A and C, upper panels), REH (B and C, lower panels), and primary B-ALL cells (D, n=6) were exposed to the indicated doses of DS with 0.5 μM Cu for 12 hr, after which mitochondrial membrane potential was measured by flow cytometry using a JC-1 kit as per the manufacturer's instruction. MFI, mean fluorescence intensity. **E.** Nalm6 cells were incubated with 0.05 μM DS +/− 0.5 μM Cu for 24 hr, followed by Western blot analysis to monitor expression of Bcl-2 and Bcl-xL, as well as cleavage of caspase 3 and PARP.

To further investigate the mechanism of action for DS/Cu to kill B-ALL cells, mitochondrial membrane potential (ΔΨm) was analyzed by flow cytometry using the ΔΨm probe JC-1 in both cultured B-ALL cell lines and primary B-ALL cells. As shown in Figure 4, treatment with DS in combination with Cu (0.5 μM) significantly reduced JC-1 up-take by Nalm6 (Figure 4A) and REH cells (Figure 4B; *P*<0.05 for DS at doses ≥ 0.05 μM, compared to untreated control, in both cell lines), of which representative data is shown in Figure 4C, consistent with induction of apoptosis (Figure 1B and 1C). Significantly, exposure (12 hrs) to DS/Cu also dose-dependently induced loss of ΔΨm in primary B-ALL cells (Figure 4D, *P*<0.05 for DS at doses ≥ 0.1 μM, compared to untreated control, n=6), isolated from the adults with B-ALL as marked in Supplemental Table S1. Furthermore, Western blot analysis revealed that whereas DS alone (24 hrs) modestly down-regulated the anti-apoptotic proteins Bcl-2 and Bcl-xL, these effects of DS was markedly enhanced in the presence of Cu, accompanied by increased caspase-3 cleavage (activation) and PARP degradation (Figure 4E). Together, these findings suggest that DS, particularly in combination with Cu, acts to induce cell death of adult B-ALL cells primarily via activation of the intrinsic apoptotic pathway, at least in part, due to down-regulation of anti-apoptotic Bcl-2 family proteins (e.g., Bcl-2 and Bxl-xL).

### DS/Cu is active *in vivo* in patient-derived xenograft (PDX) models of adult B-ALL

**Figure 5:**
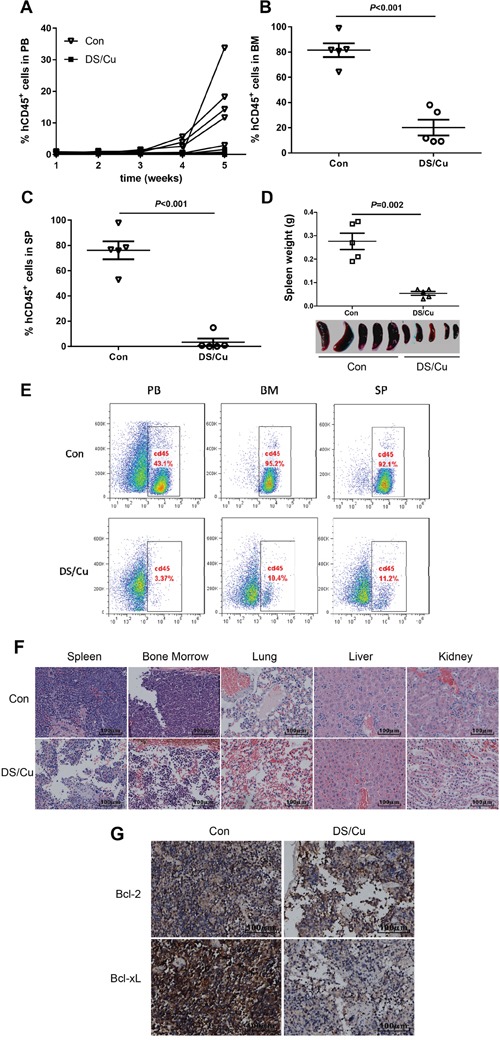
DS/Cu is active *in vivo* in patient-derived xenograft model of adult B-ALL **A-C.** Primary cells (1×10^6^ mononuclear cells per mouse) isolated from an adult with B-ALL were intravenously injected via retro-orbital vein into NSI mice. 7 days after cell inoculation, mice were randomized (n=5 per group) and treated with vehicle (control group) or DS/Cu (administered by oral gavage at dose of 1.5 mg/kg Cu in the morning and 150 mg/kg DS in the afternoon, from Monday to Friday for consecutive 4 weeks). Percentage of human CD45^+^ (hCD45) cells in peripheral blood (PB, A), bone marrow (BM, B) and spleen (SP, C) were then determined by flow cytometry. **D.** Spleens of mice were weighted and photographed at the end of the study (5 weeks after cells inoculation). **E.** Representative data of flow cytometry for detection of human CD45^+^ cells in PB, BM, and SP. **F.** Paraffin-embedded sections of spleen, bone marrow, lung, kidney, and liver were stained with H&E. **G.** Histologic sections of bone marrow were stained for human Bcl-2 and Bcl-xL by immunohistochemistry (IHC). Scale bar, 100 μm.

Last, *in vivo* anti-leukemia efficacy of DS/Cu was examined in patient-derived xenograft models of NOD-scid-IL2Rg-/- (NSI) mice, generated from the primary sample of an adult B-ALL patient with p16 deletion. Cu and DS were administered by oral gavage in the morning and afternoon respectively, from Monday to Friday for consecutive 4 weeks. Notably, mice received DS/Cu displayed a substantial delay in tumor growth, manifested by appearance of human CD45^+^ cells in peripheral blood (PB) determined by flow cytometry in none of 5 mice, while 4 of 5 mice developed CD45^+^ lesions in the control group, after 5 weeks of transplantation (Figure 5A). Consistently, co-administration of DS/Cu remarkably reduced tumor burden in the B-ALL PDX models, reflected by significantly less human CD45^+^ cells in bone marrow (BM, Figure 5B) and spleen (SP, Figure 5C) compared to control mice (*P*<0.001 for each case). Moreover, average weight (upper panel, 0.054±0.018 g for the DS/Cu group vs 0.276±0.078 g for control group, *P*=0.002) and size (lower panel) of spleens in mice received DS/Cu treatment were markedly lower or smaller than those of control mice. Representative data for detection of human CD45^+^ cells in PB, BM, and SP of PDX mice was shown in Figure 5E. Furthermore, histopathology after 5 weeks following transplantation revealed a remarkable reduction in infiltration of leukemic cells, accompanied by well preserved normal tissue architecture, in the spleen, bone marrow, lung, liver, and kidney of the PDX mice receiving DS/Cu, compared to control mice (Figure 5F). Finally, immunohistochemistry (IHC) staining demonstrated that treatment with DS/Cu clearly down-regulated the expression of Bcl-2 and Bcl-xL in bone marrow of the PDX mice (Figure 5G), consistent with the *in vitro* observation that DS/Cu activated the intrinsic apoptotic pathway (Figure 4E). Together, these findings argue strongly that the DS/Cu regimen is highly active *in vivo* in adult B-ALL PDX models.

## DISCUSSION

Evidence has been emerging on identifying new uses for existing drugs, termed repurposing or repositioning, as an accelerated way for drug development. Repositioning existing drugs could increase productivity of drug development by shortening the process from laboratory investigation to clinical application due to their easy availability and known safety or toxicity profile. DS, also known as Antabuse, has been approved by the Food and Drug Administration (FDA) for the treatment of alcohol abuse and dependence (alcoholism) for more than six decades. Recently, repositioning DS for new indications has attracted a lot of attention in treatment of cancer. The anti-cancer activity of DS, particularly in a form of coupling with Cu (DS/Cu), has been demonstrated in a variety of cancers. For example, Conticello et al. have reported that primary cells isolated from patients with various hematological malignancies, including multiple myeloma (MM), acute myeloid (AML) and lymphoblastic leukemia (ALL), were significantly sensitive to DS alone or in combination with Cu [19]. Consistently, the present studies have further validated the anti-leukemia activity of DS/Cu *in vitro* in B-ALL cell lines and especially in primary samples obtained adults with B-ALL. Of note, *in vivo* efficacy of DS/Cu was identified, to the best of our knowledge, for the first time in PDX models generated from tumor cells derived from an adult B-ALL patient.

In this pre-clinical setting, DS/Cu exhibited remarkable cytotoxicity against both B-ALL cell lines and primary adult B-ALL cells, while was only minimally toxic towards normal PBMCs obtained from healthy HSCT donors and cord blood, suggesting that the DS/Cu regimen might be able to potently and selectively eliminate adult B-ALL cells. The IC50 of DS in combination with Cu (0.5 μM) for inhibiting proliferation of Nalm6 and REH cells was 0.18 μM and 0.24 μM, respectively, which are lower than the DS IC50 of 0.5 μM in a mixture with Cu in MM cell lines [19]. It is noteworthy that the anti-proliferative IC50 of 0.5 μM for both DS and Cu is significantly lower than the concentration achieved with a normal adult dose for the treatment of alcoholism and for Cu recommended dietary intake [19]. Thus, the doses of DS and Cu that were effective against B-ALL cells fall within these low- or non-toxic dose range of these agents. Indeed, while exposure to 0.05-0.2 μM DS with 0.5 μM Cu was highly active to induce apoptosis in both B-ALL cell lines and primary adult B-ALL cells in a dose-dependent manner, treatment with the identical doses (i.e., ≤ 0.2 μM) of DS with 0.5 μM Cu displayed only minor toxicity towards normal cells. Moreover, whereas DS administrated alone had clear effects on B-ALL cells, combined treatment with non-toxic doses of Cu (e.g., 0.5 μM) dramatically enhanced activity of DS, including markedly increased induction of apoptosis and suppression of colony formation in B-ALL cells. Moreover, the latter also provides evidence for the capability of DS/Cu to impair clonogenicity of B-ALL cells. Notably, DS/Cu was also highly active *in vivo* in PDX models of adult B-ALL, which were developed using humanized NSI mouse [25, 26], including substantial delay of tumor growth, reduction of tumor burden, and attenuation of leukemic cell infiltration in multiple organs (e.g., spleen, bone marrow, lung, liver, and kidney). Taken together, these *in vitro* and *in vivo* findings argue strongly that DS, especially when administrated in combination with Cu, might represent an effective repurposing regimen for the treatment of adult B-ALL.

It is well established that the therapeutic response and prognosis of patients with ALL are significantly influenced by multiple risk factors, including age, WBC count at the time of first diagnosis, extramedullary infiltration, cytogenetic abnormalities, etc. [27–31] Moreover, high-risk diseases are associated with greater incidence of chemo-resistance, higher rate of relapse, and lower rate of survival. To this end, we further analyzed the relationship between clinical characteristics and anti-tumor activity of DS/Cu in their primary samples obtained from 32 adults with B-ALL. Interestingly, we found that the *ex vivo* cytotoxicity of DS/Cu was significantly associated with peripheral WBC count at diagnosis and p16 gene deletion, but not other clinical features such as age, LDH value, extramedullary infiltration, status at day 14 and 28 after intensive induction therapy, immune phenotype, risk category, and Ph chromosome. While the mechanisms underlying such correlations between therapeutic responses to DS/Cu and WBC counts or p16 deletion remain to be explored in successor studies, these findings might provide initial evidence for future development of the DS/Cu regimen as personalized treatment of adult B-ALL.

Several mechanisms have been reported for induction of apoptosis by DS/Cu in cancer cells, including production of reactive oxygen species [10], inhibition of proteasome activity [11], regulation of transcription factors (e.g., NF-κB), and activation of the stress-related JNK signaling pathway [17, 18]. It has also been demonstrated that DS/Cu cytotoxicity may attribute to modulation of the anti- and pro-apoptotic Bcl-2 family proteins in human glioblastoma cells, as well as ALDH-positive cancer stem-like cells [10] and breast cancer stem cells [12]. The results of the present study elucidate that DS/Cu induced apoptosis of B-ALL cells most likely via activation of the mitochondria-related intrinsic apoptotic pathway, reflected by loss of mitochondria membrane potential, down-regulation of anti-apoptotic Bcl-2 family proteins (e.g., Bcl-2 and Bcl-xL), and following caspase-3 activation and PARP degradation. A similar mechanism for anti-leukemia activity of DS/Cu might also operate *in vivo* in PDX models of adult B-ALL, manifested by down-regulation of Bcl-2 and Bcl-xL after co-administration of DS/Cu. While the mechanisms underlying down-regulation of these anti-apoptotic proteins by DS/Cu remains to be defined, the results of the present study suggest that DS/Cu might primarily act to activate the mitochondria-mediated intrinsic apoptotic pathway, at least in part, via down-regulation of the anti-apoptotic Bcl-2 family proteins (e.g., Bcl-2 and Bcl-xL).

In summary, the present study demonstrated that DS in combination with Cu effectively and selectively eradicates adult B-ALL cells, including inhibition of tumor cell proliferation, induction of apoptosis, impair of clonogenicity, and suppression of tumor growth in PDX models. Significantly, cytotoxicity of DS/Cu correlated to certain clinical adverse prognostic factors including WBC counts at diagnosis and p16 gene deletion. Moreover, anti-leukemia activity of DS/Cu was mechanistically associated with down-regulation of the anti-apoptotic Bcl-2 family members and activation of the mitochondria-mediated intrinsic apoptotic pathway. Therefore, the present findings suggest that the DS/Cu regimen warrants further consideration in adult B-ALL, particularly certain clinical/genetic subsets with poor prognosis.

## MATERIALS AND METHODS

### Chemicals and reagents

Disulfiram (Santa Cruz Biotechnology Inc., Santa Cruz, CA, USA) and copper (Sigma-Aldrich, Dorset, UK) were dissolved in DMSO as 5 mM stock solution and phosphate-buffered saline (PBS) as 100 mM stock solution, respectively. Both stock solutions were stored at −20°C and freshly diluted with culture medium before use.

### Cell lines and cell culture

Nalm6 and REH cell lines were purchased from ATCC (Teddington, UK). Cells were cultured in RPMI-1640 (HyClone, Thermo Scientific, Waltham, MA, USA) supplemented with 10% fetal bovine serum (FBS, Gibco, Life Technologies, NY, USA), 100 U/ml penicillin and 100μg/ml streptomycin (1×P/S).

### Primary samples

Peripheral blood (PB) samples from healthy hematopoietic stem cell transplantation (HSCT) donors and bone marrow (BM) samples from newly diagnosed adult B-ALL patients were obtained at Department of Hematology, Nanfang Hospital, Southern Medical University. Informed consent was provided for research purposes only, approved by the Nanfang Hospital Ethics Review Board, in accordance with the Declaration of Helsinki. Clinical characteristics of the B-ALL patients are summarized in Table 1 and Supplemental Table S1. Mononuclear cells were isolated by density gradient centrifugation using Lymphoprep^TM^ (BD, Franklin Lakes, NJ, USA), washed twice with PBS, and cultured in IMDM (HyClone, Thermo Scientific) supplemented with 10% FBS (Gibco, Life Technologies), 100 U/ml penicillin and 100μg/ml streptomycin (1×P/S).

### Cell counting kit-8 (CCK-8) assay

Cytotoxicity of DS/Cu was determined using a CCK-8 kit (Dojindo, Kumamoto, Japan). Briefly, cells (5×10^4^ cells/well) were plated into 96-well plates containing 100 μl of growth medium and then treated with designated doses of DS in combination with 0.5 μM Cu for 24 hrs. After treatment, CCK-8 reagents (10 μl/well) were added and incubated for 2 hrs at 37°C in a 5% CO_2_ incubator. Finally, absorbance at 450 nm were read by a microplate reader (ELX800, Bio TEK, USA). All experiments were repeated three times and performed in triplicate in each experiment. The IC_50_ value of each cell line was calculated using GraphPad Prism 5.

### Annexin V-APC/PI double staining assay by flow cytometry

Apoptosis was measured using Annexin V-APC/PI (Ebioscience, San Diego, USA) dual staining by flow cytometry. Briefly, cells (2×10^5^/well) were seeded into 24-well plates and exposed to DS at different doses (0.025, 0.05, 0.1, 0.2 μM) either with or without Cu (0.5 μM) for 24 hrs. Cells were harvested, washed twice with iced PBS, and double labeled with Annexin V-APC/PI for 30 minutes at 4°C in the dark. Cells were then analyzed by flow cytometry using FACS C6 (BD, Oxford, UK).

### Clonogenic assay

Cells (2×10^5^/well) were seeded in 24-well plates and treated for 6 hrs with DS (0.05 μM) alone or in combination with Cu (0.5 μM). Cells were collected and further cultured in complete methylcellulose medium at a cell density of 500/well in 6-well plates for 14 days. Colonies consisting of at least 50 cells were counted and analyzed for clonogenicity.

### Analysis of mitochondrial membrane potential

Mitochondrial membrane potential (MMP, ΔΨm) was determined using a JC-1 kit (Beyotime Biotechnology, China) as per the manufacturer's instruction. After exposed to various concentrations of DS in combination with 0.5 μM Cu for 12 hrs, cells were collected, washed twice with iced PBS, resuspended in 500 μl 1× working JC-1 solution, and incubated for 20 min at 37°C in the dark. After washing twice with a JC-1 buffer solution, MMP were analyzed by FACS C6 (BD).

### Western blot analysis

Whole cell lysates (30 μg protein/lane) was electrophoresed in 10% SDS- PAGE and transferred to a PVDF membrane (Millipore, Billerica, MA, USA). After blocked for 1 hr for non-specific binding in TBS-T with 5% non-fat milk, blots were incubated with primary antibodies (Bcl-2, rabbit polyclonal, 1:500; PARP, rabbit polyclonal, 1:500; Bcl-xL, rabbit polyclonal, 1:1000, Cell Signaling Technology, Inc. Herts, UK; caspase-3, rabbit monoclonal, 1:1000, Beyotime, China) overnight at 4°C, followed by secondary HRP-conjugated monoclonal antibody (1:10000, Cell Signaling Technology) for 1 h at room temperature. Blots were re-probed with β-actin antibody (rabbit-monoclonal, 1:1000, Cell Signaling Technology) to ensure equal protein loading. Blots were visualized on X-ray films using an enhanced chemiluminescence Western blotting detection kit (Amersham, Little Chalfont, UK).

### Animal studies in patient-derived xenograft (PDX) models

Male NSI 6 to 8 week old mice [25, 26] were kindly provided by Dr. Peng Li (Guangzhou Institutes of Biomedicine and Health, Chinese Academy of Sciences, Guangzhou, China) and housed under pathogen-free conditions according to the animal care guidelines. The protocols for the animal studies were approved by Southern Medical University. On the day of tumor cell inoculation, mice received 1 Gy of total body irradiation at a dose rate of 325 cGy/min by parallel opposed 4 MV x-ray. Within 24 hrs, mice were intravenously injected via retro-orbital vein with 1×10^6^ primary mononuclear cells isolated from adults with B-ALL. Seven days after transplantation of patient B-ALL cells, mice were randomly assigned to either control or DS/Cu group (n = 5 per group) and then treated respectively with either vehicle (PBS and 0.5% methyl cellulose/0.5% Tween 80 in PBS) or 1.5 mg/kg/d Cu in the morning followed by 150 mg/kg/d DS in the afternoon by oral gavage from Monday to Friday for 4 consecutive weeks. Tumor burden of mice was monitored every 7 days by flow cytometry after staining peripheral blood (PB, 50-100 μL) collected from retro-orbital vein with an anti-human-CD45 antibody. At the end of the experiments, mice were euthanized, after which their spleens were photographed and weighed, and leukemia load (human CD45^+^ cells) in peripheral blood (PB), bone marrow (BM) and spleen was determined by flow cytometry after staining with anti-human-CD45 antibody. The viscera organs (including BM, spleen, liver and kidney) were removed and fixed in 10% paraformaldehyde for 24 hrs, after which paraffin-embedded sections were prepared and subjected to H&E staining and immunohistochemistry (IHC) for human Bcl-2 and Bcl-xL.

### Statistical analysis

Values represent the mean ±SD for at least three independent experiments. Comparisons between two groups were analyzed using the 2-tailed Student's t test. Multiple-group comparisons were performed using the One-way analysis of variance (ANOVA) followed by the Bonferroni posthoc test. Two-way ANOVA analysis was used to examine the relationship between various clinical characteristics of B-ALL patients and cytotoxicity of DS/Cu against primary B-ALL cells. Analyses utilized IBM SPSS 19.0 and GraphPad Prism 5.0 software. *P* values < 0.05 was considered as statistically significant.

## SUPPLEMENTARY TABLE


