# Supplementary material for: Effective elimination of adult B-lineage acute lymphoblastic leukemia by disulfiram/copper complex in vitro and in vivo in patient-derived xenograft models
Source: Oncotarget. 2016 May 17;7(50):82200–12. doi: 10.18632/oncotarget.9413 (PMC5347685; doi:10.18632/oncotarget.9413)
Supplement: Supplementary file 1 [file oncotarget-07-82200-s001.pdf]

SUPPLEMENTARY TABLE

Supplementary Table S1: Clinical characteristics of B-lineage acute lymphoblastic leukemia patients in this study

No.	Sex	Age (yr)	WBC at biopsy (*10^9/L)	LDH (U/L)	Extramedullary infiltration	Status of d14	Status after induction therapy	Risk category	Immune phenotype	Ph chromosome	p16 gene deletion
1	M	18	93.69	892.4	Yes	CR	CR	Standard	Non-proB	Negative	Positive
2	M	34	78.23	1833	No	NR	NR	High	Non-proB	Positive	Positive
3	M	15	429.46	7630	Yes	CR	CR	Standard	Pro-B	Negative	Positive
4	M	43	36.52	149	No	CR	CR	High	Non-proB	Positive	Positive
5	M	40	295.37	810.9	Yes	NR	CR	High	Pro-B	Positive	Negative
6	M	31	3.88	176	Yes	NR	NR	Standard	Non-proB	Negative	Negative
7	M	28	67.99	939	Yes	NR	NR	High	Pro-B	Positive	Negative
8	M	36	83.1	>10000	Yes	CR	CR	Standard	Non-proB	Negative	Positive
9	M	61	136.51	1965.5	Yes	CR	CR	High	Non-proB	Positive	Positive
10[a]	M	20	46.3	247.5	Yes	NR	CR	Standard	Non-proB	Negative	Negative
11	F	19	24.48	22488	Yes	CR	CR	Standard	Non-proB	Negative	Positive
12	M	42	105.91	1135.6	Yes	CR	CR	High	Non-proB	Positive	Positive
13	F	25	1.27	207.8	Yes	CR	CR	Standard	Non-proB	Negative	Positive
14	F	19	237.8	1164.6	Yes	NR	NR	Standard	Pro-B	Negative	Positive
15[a]	M	21	4.9	213.7	Yes	NR	NR	Standard	Pro-B	Negative	Negative
16	M	18	326.13	501.1	Yes	NR	CR	High	Pro-B	Positive	Negative
17	M	35	64,82	184.6	No	NR	NR	High	Pro-B	Negative	Negative
18	M	33	24.86	407	Yes	CR	CR	Standard	Pro-B	Negative	Negative
19[a]	M	35	33.5	137.4	No	CR	CR	High	Pro-B	Positive	Negative
20	M	27	33	4165.4	Yes	CR	CR	High	Non-proB	Positive	Negative
21	M	56	7.48	1738	Yes	NR	CR	Standard	Non-proB	Negative	Negative
22	M	25	38.88	109	No	CR	CR	Standard	Pro-B	Negative	Negative
23	M	19	50.62	608	No	NR	NR	High	Non-proB	Positive	Negative
24[a]	M	32	46.83	152.5	No	CR	CR	Standard	Non-proB	Negative	-
25[a]	F	17	12.66	2918	Yes	CR	CR	Standard	Non-proB	Negative	Positive
26	F	16	68.32	216.3	Yes	CR	CR	Standard	Pro-B	Negative	-
27	M	42	34.19	484	Yes	CR	CR	High	Non-proB	Positive	Negative
28	M	22	153.91	1285.5	No	CR	CR	Standard	Non-proB	Negative	-
29	M	47	4.47	3307	Yes	NR	CR	High	Non-ProB	Negative	Negative
30	M	18	11.89	356	Yes	CR	CR	Standard	Pro-B	Negative	Negative
31	F	37	9.2	614	Yes	CR	CR	Standard	Non-proB	Negative	Positive
32[a]	M	18	323.5	1135.2	Yes	CR	CR	Standard	Pro-B	Negative	-

Abbreviations: F, female; M, man; WBC, white blood cell; LDH, lactic dehydrogenase; CR, complete remission; NR, No remission; [a] the samples used for analysis of mitochondrial membrane potential.
